# Optimized Anti-pathogenic Agents Based on Core/Shell Nanostructures and 2-((4-Ethylphenoxy)ethyl)-*N*-(substituted-phenylcarbamothioyl)-benzamides

**DOI:** 10.3390/ijms131012584

**Published:** 2012-10-01

**Authors:** Carmen Limban, Alexandru Mihai Grumezescu, Crina Saviuc, Georgeta Voicu, Gentiana Predan, Robert Sakizlian, Mariana Carmen Chifiriuc

**Affiliations:** 1 Department of Pharmaceutical Chemistry, “Carol Davila” University of Medicine and Pharmacy, Traian Vuia no.6, 020956 Bucharest, Romania; E-Mail: carmen_limban@yahoo.com; 2 Department of Science and Engineering of Oxidic Materials and Nanomaterials, Faculty of Applied Chemistry and Materials Science, University Politehnica of Bucharest, Polizu Street no 1-7, 011061 Bucharest, Romania; E-Mail: getav2001@yahoo.co.uk; 3 Department of Microbiology, Faculty of Biology, Universtity of Bucharest, Aleea Portocalelor no. 1-3, 060101 Bucharest, Romania; E-Mails: carmen_balotescu@yahoo.com (M.C.C.); crina.saviuc@yahoo.com (C.S.); ggentiana@yahoo.com (G.P.); 4 Department of Physical Education and Sport, University of Bucharest, Bvd. M. Kogalniceanu 36-46, Bucharest, Romania; E-Mail: sakizlian@yahoo.com

**Keywords:** benzamides, thiourea derivatives, core/shell nanostructure, magnetite, anti-biofilm, biointerface application

## Abstract

The purpose of this study was to design a new nanosystem for catheter surface functionalization with an improved resistance to *Staphylococcus aureus* ATCC 25923 and *Pseudomonas aeruginosa* ATCC 27853 colonization and subsequent biofilm development. New 2-((4-ethylphenoxy)methyl)-*N*-(substituted-phenylcarbamothioyl)-benzamides were synthesized and used for coating a core/shell nanostructure. Their chemical structures were elucidated by NMR, IR and elemental analysis, being in agreement with the proposed ones. Fe_3_O_4_/C_12_ of up to 5 nm size had been synthesized with lauric acid as a coating agent and characterized by XRD, FT-IR, TGA, TEM and biological assays. The catheter pieces were coated with the fabricated nanofluid in magnetic field. The microbial adherence ability was investigated in 6 multiwell plates by using culture based methods and Scanning Electron Microscopy (SEM). The nanoparticles coated with the obtained compounds **1a**–**c** inhibited the adherence and biofilm development ability of the *S. aureus* and *P. aeruginosa* tested strains on the catheter functionalized surface, as shown by the reduction of viable cell counts and SEM examination of the biofilm architecture. Using the novel core/shell/adsorption-shell to inhibit the microbial adherence could be of a great interest for the biomedical field, opening new directions for the design of film-coated surfaces with improved anti-biofilm properties.

## 1. Introduction

The compounds containing thiourea function in their molecules are suitable candidates as pharmacologically active compounds and also they have a long history of being used as ligands in coordination chemistry. Thiourea derivatives have found extensive applications in the field of medicine and agriculture. They are known to exhibit a wide variety of biological activities such as antimycobacterial [1], anthelmintic [2], antimalarial [3], antitumor [4], analgesic [5] bioactivities and inhibitory activities against viruses [6], and also useful as insecticides [7], fungicides [8], herbicides [9], and plant-growth regulators [10]. Thiourea derivatives have also been reported to possess antibacterial and antifungal properties. Gülkok *et al.* have synthesized new compounds containing a thiourea group at position 6 of 3-methyl-2(3*H*)-benzoxazolone and 5-chloro-3-methyl-2(3*H*)-benzoxazolone rings and investigated their antibacterial and antifungal activities. *Escherichia coli* and *Enterococcus faecalis* were generally more susceptible to these compounds in comparison with the other used bacteria [11]. Some new derivatives of 1-(4-(3-(4-methoxyphenyl)thioureido)-6-(1*H*-1,2,4-triazol-1-yl)-1,3,5-triazin-2-yl)-3-phenylurea, having three components coupled to triazole, for enhancing the biological activity, were synthesized and evaluated for their *in vitro* antimicrobial activity. One of these compounds exhibited excellent activity against *Bacillus subtilis*, *Pseudomonas aeruginosa* and *Candida albicans* and three of them showed excellent activity against *Staphylococcus aureus* [12]. Reddy *et al.* described the synthesis, spectroscopic identification and antibacterial activity of some novel thiourea derivatives at C-8 alkyl chain of anacardic acids against *E. coli*, *P. aeruginosa*, *S. aureus* and *Streptococcus pyogenes* bacterial strains. Some of the tested compounds showed a more intensive biological activity than the used antibiotic standard [13].

1-Phenyl-3-[1-cyclopropyl-6-fluoro-7-((un)substituted-piperazin-1-yl)-4-oxo-1,4-dihydro-quinoline-3-carbonyl]thiourea derivatives were synthesized and evaluated for their antibacterial activity against *S. aureus*, *B. subtilis*, *E. coli*, and *P. aeruginosa* and compared with the standard drug ciprofloxacin. Significant improvement of these thioureido amide fluoroquinolone activity was seen when compared with previously synthesized esters and amides in C-3 position of fluoroquinolones [14]. A new series of 2-(6′-fluorobenzothiazol-2′-ylamino)-4,6-(disubstituted thioureido)-1,3-pyrimidine derivatives have been synthesized and screened for their *in vitro* growth inhibiting activity against different bacterial strains of *E. coli*, *P. aeruginosa*, *S. aureus* and *B. subtilis*, using the agar diffusion technique, the compounds having fluoro or nitro groups in the molecule displaying good activity [15]. Modi *et al*. found that the 2-oxo-4,6-diphenyl-2,6-dihydropyrazolo[1,5-α][1,3,5]triazine-7 carbonyl ring system with thiourea showed good antibacterial activity against *S. aureus* [16]. 6-*tert*-Butyl-4-(4-R-6-morpholino-1,3,5-triazin-2-yl)-3-(methylthio)-1,2,4-triazin-5(4*H*)-one (R = substituted thiourea) were prepared using the substitution of chlorine in 2,4,6-trichloro-*s*-triazine by some moieties having structural as well as biological importance. They were subjected to antibacterial and antifungal screening and showed excellent activity against *S. aureus*, *B. subtilis*, *E. coli*, *P.aeruginosa*, and *C. albicans* [17]. 3,4-Dimetoxiphenylethyl-1,3,5-triazynil thiourea derivatives, prepared by condensation of 2,4,6-trichloro-1,3,5-*s*-triazine with 4-hydroxycoumarin, 3,4-dimethoxyphenylethylthiourea and various substituted phenylthiourea, were tested for their antibacterial and anti-HIV activities and showed comparable activity with the standard drugs against *S. aureus*, *Salmonella typhi*, *E. coli* and *B. subtilis* [6]. A set of substituted piperazinyloxazolidinone derivatives has been studied for their antibacterial activity against some Gram-positive bacteria. Substituting the acetamide group at 5-position of linezolid, the only drug of this class in the market, by a thiourea group, the antibacterial activity was improved [18].

(*S*)-*N*′-Benzoyl-*N*-[[3-[3-fluoro-4-(1-pyrrolidinyl)phenyl]-2-oxo-5-oxazolidinyl]-methyl]thiourea and (*S*)-*N*-[[3-[3-fluoro-4-(1-pyrrolidinyl/substituted-1-piperidinyl)-phenyl]-2-oxo-5-oxazolidinyl] methyl]thiourea derivatives were prepared for SAR study, especially the relationship between lipophilicity and antibacterial activity against Gram-positive bacteria including methicillin-resistant *S. aureus* (MRSA) and vancomycin-resistant *Enterococcus* sp. (VRE) [19]. 1-[3-[*N*-[3-[3-(piperidinomethyl)phenoxy]propyl]carbamoyl]propyl]-3-alkyl/cycloalkyl/aralkyl/substituted aryl/heteroarylthiourea derivatives are useful as a therapeutic agents for peptic ulcers, having high gastric anti-secretory and defense factor-reinforcing activities, which is also effective on prevention of the recurrence after discontinuation of the administration due to the antimicrobial activity against *Helicobacter pylori*, which could prevent the recurrence of peptic ulcers [20].

*S. aureus* is a versatile human pathogen which has the ability to cause a wide variety of human diseases, ranging from skin lesions such as abscesses and impetigo to invasive and more serious infections such as osteomyelitis, septic arthritis, pneumonia, and endocarditis [21,22]. According to EARSS data, susceptibility results for *S. aureus* MRSA isolates in Romania varies largely from 36.3% in 2002 and 71.7% in 2004 to 54.2% in 2006 and 26.2% in 2007 [23]. The initial step in the pathogenesis of *S. aureus* infection is the attachment of the organism to the human cell surfaces and implanted devices, mediated by a series of bacterial adhesins.

*P. aeruginosa* is an opportunistic pathogen that causes many types of infections: urinary tract infections, respiratory system infections, dermatitis, soft tissue infections, bacteremia, bone and joint infections, gastrointestinal infections and a variety of systemic infections, particularly in patients with severe burns and in cancer and AIDS patients who are immunosuppressed. The *P. aeruginosa* strains isolated in Romania from invasive infections showed high resistance rates to cephtazidime (52.2%) and to carbapenems (60.9%) [24].

An important feature of both *S. aureus* and *P. aeruginosa* pathogenesis is their ability to form surface-associated, structured and cooperative consortia referred to as biofilms, which play an important role in bacterial pathogenesis and is a common cause of persistent infections. Bacteria from biofilm are resistant to disinfectants, antibiotics and the action of host immune defenses [24].

Nanosized magnetite is one of the most important material and it is widely used in biomedical field [25,26]. Medical applications like drug targeting [27,28], inhibition of microbial biofilm development [29], magnetic resonance imaging [30,31] or hyperthermia [32] are really exciting and are under development at the moment.

In the present work, we report the fabrication, characterization of novel core/shell/adsorption-shell for catheter surface functionalization to resist microbial colonization and prevent the *S. aureus* and *P. aeruginosa* biofilm development.

## 2. Results and Discussion

### 2.1. Synthesis and Chemical Characterization

**Scheme 1:**
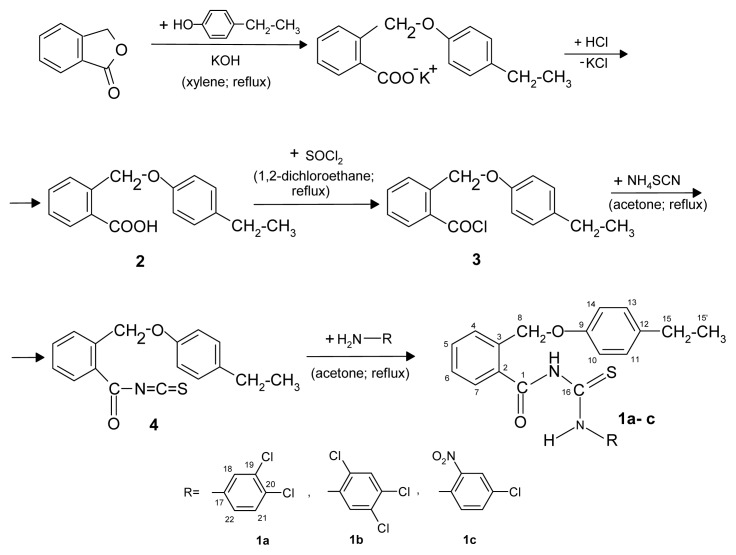
The pathway for the synthesis of the new thioureides.

The new thioureides **1a**–**c** were synthesized in good yields by a series of reactions as shown in Scheme 1.

The new thioureides are white or light yellow crystalline solids, soluble at room temperature in chloroform and acetone, by heating in lower alcohols, benzene, toluene and xylene and insoluble in water. The new compounds have been characterized by their melting point, elemental analysis (Table 1), IR and NMR spectral studies.

All spectroscopic and elemental analyses data confirm the proposed structures of the new compounds. The IR bands were given as w—weak, m—medium, s—strong, vs—very strong and were obtained using attenuated total reflection Fourier transformed infrared (FT-IR ATR) spectra at room temperature.

The structure of the new compounds is also supported by NMR spectra. The new thioureides were dissolved in dmso-d6 (hexadeuteriodimethyl sulphoxide) and the chemical shifts values, expressed in parts per million (ppm) were referenced downfield to tetramethylsilane, for ^1^H-NMR and ^13^C-NMR and the constants (*J*) values in Hertz. The chemical shifts for hydrogen and carbon atoms were established also by GCOSY, GHMBC, GHSQC experiments. The ^1^H-NMR data are reported in the following order: chemical shifts, multiplicity, the coupling constants, number of protons and signal/atom attribution. Spin multiplets are given as s (singlet), d (doublet), t (triplet), q (quartet), m (multiplet), dd (double doublet), td (triple doublet), and br (broad) signal. For the ^13^C-NMR data the following order are: chemical shifts and signal/atom attribution in some cases.

The culture based method demonstrated that the incorporation of the chemical substances **1a**–**c** in the nanosystem, improved the antibiofilm activity of all three compounds.

**Figure 1:**
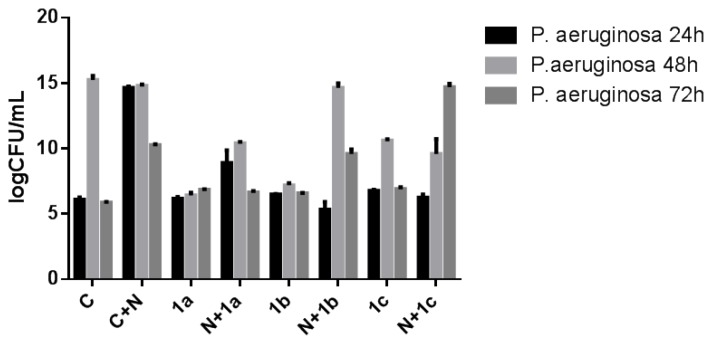
The viable cell counts recovered from *P. aeruginosa* biofilms developed on the catheter pieces harvested at different incubation times (C+N—catheter coated with nanoparticles; C—catheter control; 1a, 1b, 1c—catheter pieces immersed in pure chemicals solutions without being attached to the nanoparticles; N+1a, N+1b, N+1c—catheter pieces immersed in pure chemicals solutions attached to the nanoparticles).

In case of *P. aeruginosa* biofilms, at 24 h the catheter coated with nanoparticles proved to be generally more susceptible to the microbial colonization, as compared to the uncoated catheter. All tested soluble compounds, as well as those included into nanoparticles induced a statistically significant improvement of the anti-biofilm activity of the catheter sections as compared with the nanoparticles coating, when tested at 24 and 48 h (*p* < 0.0001) (Figure 1), the most efficient being the compounds **1a** and **1c**. It is well known that *P. aeruginosa* is naturally resistant to different antimicrobial substances. A major contribution to this intrinsic multidrug resistance is provided by a number of broadly-specific multidrug efflux systems, which in addition to antibiotics, promote export of numerous dyes, detergents, inhibitors, disinfectants, organic solvents and signaling molecules [33].

At 72 h, the compound **1a** included in the nanoparticles layer proved to be significantly more efficient against biofilm colonization than the uncoated catheter (*p* < 0.0001). It is thus clear that the incorporation of the tested compounds in the nanoparticles layer may improve the release of the antimicrobial substance and its accumulation in active concentrations at the level of the bacterial target.

**Figure 2:**
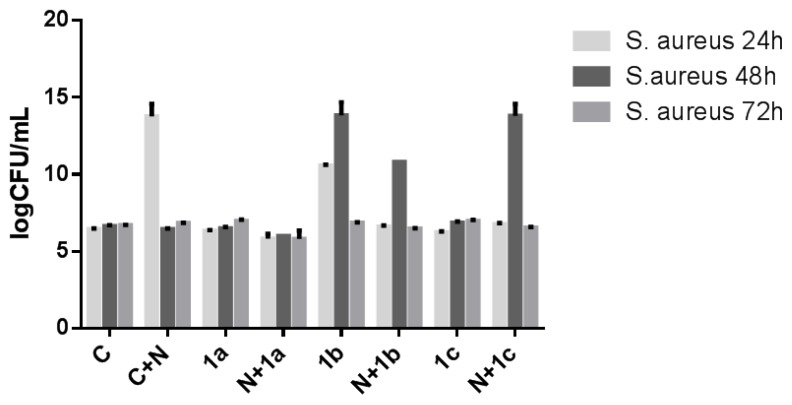
The viable cell counts recovered from *S. aureus* biofilms developed on the catheter pieces harvested at different incubation times (C+N—catheter coated with nanoparticles; C—catheter control; 1a, 1b, 1c—catheter pieces immersed in pure chemicals solutions without being attached to the nanoparticles; N+1a, N+1b, N+1c—catheter pieces immersed in pure chemicals solutions attached to the nanoparticles).

In case of *S. aureus* biofilms, the same susceptibility of the catheter sections coated only with nanoparticles layer, as compared to the uncoated catheters or those coated with naoparticles and soluble compounds, has been obtained at 24 h (*p* < 0.0001). The inclusion of the tested compounds into nanoparticles was associated with the occurrence of an intensive anti-biofilm effect at 24 h (*p* < 0.0001) as compared with the catheter coated only with nanoparticles. At 48 h, the compound **1a** both in soluble form and included into nanoparticles and **1c** in soluble form, exhibited antibiofilm activity superior to that of uncoated catheter sections or of catheter coated only with nanoparticles. At 72 h only the compound **1a** included in the nanosystem exhibited inhibitory activity (Figure 2).

**Figure 3:**
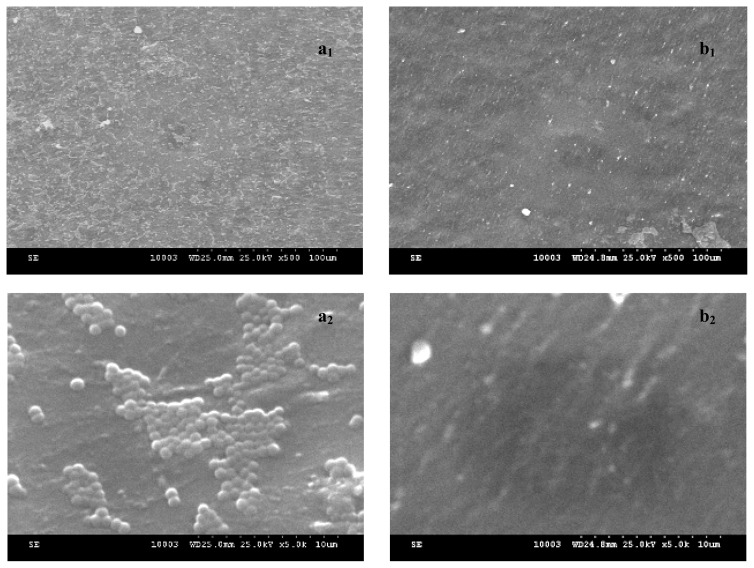
*S. aureus* biofilm architecture examined by Scanning Electron Microscopy (SEM) for (**a**) uncoated and (**b**) coated (core/shell/**1b**) prosthetic devices after 24 h of incubation (**a_1_**—*S. aureus* biofilm developed on the untreated catheter surface, covering homogenously the entire surface; **a_2_**—*S. aureus* colonies developed on the untreated catheter surface; **b_1_**—rare microbial cells adhered to the surface of the functionalized catheter; **b_2_**—functionalized catheter surface without any adhered bacterial cells).

Our quantitative assay has clearly demonstrated that the inclusion of the tested chemical substances into the core/shell nanosystem induced and/or improved their anti-biofilm activity. These results have been also confirmed by the qualitative assay of the microbial biofilms developed on the catheter surfaces by SEM (Figure 3). The SEM images performed on the *S. aureus* biofilms developed on the catheter control and on the functionalized one show a mature biofilm developed on the surface of the untreated catheter, while on the functionalized catheter surface the microbial cells were very rare or even absent (Figure 3).

## 3. Experimental Section

### 3.1. Synthesis and Characterization of Core/Shell Nanostructure

Core/shell nanostructure was prepared and characterized according to our recently published paper [32]. Briefly, lauric acid was solubilized in a known volume of distilled-deionized water, corresponding to a 1.00% (*w*/*w*) solution, under stirring at room temperature. Then, basic aqueous solution consisting of 28% NH_3_ was added to lauric acid solution. After these, FeSO_4_/FeCl_3_ (1:2 molar ratio) were dropped under permanent stirring up to pH = 8, leading to the formation of a black precipitate. The product was repeatedly washed with methanol and separated with a strong NdFeB permanent magnet. In our previous study [34] we report the successfully fabrication of core/shell nanostructure and its characterization by XRD, TEM and FT-IR. The obtained powder was identified as magnetite by XRD. Dimension of core/shell structure not exceeding 5 nm and their spherical shape was confirmed by TEM analysis. The FT-IR analysis identified the organic coating agent, *i.e.* lauric acid on the surface of the magnetite nanoparticles. Treatment for 24 h with Fe_3_O_4_/C_12_ seems to not be cytotoxic on the HEp2 cell line, this aspect representing an advantage for the *in vivo* use of these nanostructure systems for biomedical applications with minor risks for the occurrence of side effects [34].

### 3.2. Synthesis and Characterization of Adsorption-Shell

#### 3.2.1. Chemistry

All chemicals used for the synthesis of the new compounds were purchased from Merck, Sigma-Aldrich or Fluka companies. *para*-Ethylphenol was used freshly distilled. Acetone was dried over K_2_CO_3_ and then distilled and ammonium thiocyanate was treated by heating at 100 °C before use. Melting points were recorded on a Electrothermal 9100 capillary melting point apparatus in open capillary tubes and the values are uncorrected.

Microanalysis for carbon, hydrogen, nitrogen and sulfur was carried out with a Perkin Elmer CHNS/O Analyzer Series II 2400 and the results being within ±0.4% of the theoretical values. The FT-IR spectra of the all synthesized compounds were run on a Bruker Vertex 70 spectrophotometer. The NMR spectra were performed on a Varian Unity Inova 400 instrument operating at 400 MHz for ^1^H and 100 MHz for ^13^C. Starting compounds for this synthesis, the 2-(4-ethyl-phenoxymethyl)benzoic acid (**2**) and the 2-(4-ethyl-phenoxymethyl)benzoyl chloride (**3**) have prevopisly been prepared in good yields [35].

#### 3.2.2. General Synthesis Procedure of the New Thioureides

A solution of 2-(4-ethylphenoxymethyl)benzoyl chloride (**3**) (0.01 mol) in acetone (15 mL) was added to a solution of ammonium thiocyanate (0.01 mol) in acetone (5 mL) to afford arylisothiocyanate **4** *in situ*. The reaction mixture was heated under reflux for 1 h, and then cooled at the room temperature. A solution of primary amine (0.01 mol) in acetone (2 mL) was added to the mixture and heated under reflux for 1 h. The acylthioureas was precipitated after the cooled reaction mixture was poured into 500 mL water. The solid product was purified by recrystallization from isopropanol with active carbon.

#### 3.2.3. Characterization of Coated-Shell

##### 3.2.3.1. 2-((4-Ethylphenoxy)methyl)-*N*-(3,4-dichlorophenylcarbamothioyl)benzamide (1a)

^1^H-NMR (dmso-d6, δ ppm): 12.40 (s, 1H, NH); 11.96 (br s, 1H, NH); 7.98 (d, *J* = 2.1 Hz, 1H, H-18); 7.65 (d, *J* = 8.6 Hz, 1H, H-21); 7.61 (br d, *J* = 7.4 Hz, 1H, H-7); 7.60–7.54 (m, 2H, H-4, H-5); 7.54 (dd, *J* = 2.1 Hz, *J* = 8.6 Hz, 1H, H-22); 7.47 (td, *J* = 1.4 Hz, 7.5 Hz, 1H, H-6); 7.08 (d, *J* = 8.6 Hz, 2H, H-11, H-13); 6.89 (d, *J* = 8.6 Hz, 2H, H-10, H-14); 5.26 (s, 2H, H-8); 2.51 (q, *J* = 7.5 Hz, 2H, H-15); 1.15 (t, *J* = 7.5 Hz, 3H, H-15′).

^13^C-NMR (dmso-d6, δ ppm): 179.44 (C-16); 170.00 (C-1); 156.26 (C-9); 137.97; 136.18; 135.84; 133.20; 131.04 (C-5); 130.62; 130.03 (C-20); 128.55 (C-11, C-13); 128.16; 128.42 (C-4); 128.34 (C-7); 127.71 (C-6); 126.03 (C-18); 124.85 (C-22); 114.52 (C-10, C-14); 67.47 (C-8); 27.22 (C-15); 15.71 (C-15′).

FT-IR (ν, cm^−1^): 3333m (νN-H of amide group); 3098w (νN-H of thioamide group); 2957m (antisymmetric vibration νC-H of methyl group); 2927m (antisymmetric vibration νC-H of methylene group); 2870m; 1678m (νC = O); 1602m; 1581s; 1505vs (δN-H of amide group); 1471s; 1384m; 1336s; 1296m; 1223s (antisymmetric vibration for alkyl-aryl-eter); 1173w (νC = S); 1138s; 1040m; 1024s (symmetric vibration for alkyl-aryl-eter); 877w; 820w; 779w; 737m; 708w; 667m; 607w.

##### 3.2.3.2. 2-((4-Ethylphenoxy)methyl)-*N*-(2,4,5-trichlorophenylcarbamothioyl)benzamide (1b)

^1^H-NMR (dmso-d6, δ ppm): 12.53 (br s, 1H, NH); 12.18 (br s, 1H, NH); 8.31 (s, 1H, H-19); 7.96 (s, 1H, H-22); 7.62 (br d, *J* = 7.4 Hz, 1H, H-7); 7.59 (m, 1H, H-4); 7.57 (td, *J* = 1.4 Hz, *J* = 7.4 Hz, 1H, H-5); 7.47 (td, *J* = 1.4 Hz, *J* = 7.5 Hz, 1H, H-6); 7.08 (d, *J* = 8.6 Hz, 2H, H-11, H-13); 6.89 (d, *J* = 8.6 Hz, 2H, H-10, H-14); 5.26 (s, 2H, H-8); 2.51 (q, *J* = 7.5 Hz, 2H, H-15); 1.11 (t, *J* = 7.5 Hz, 3H, H-15′).

^13^C-NMR (dmso-d6, δ ppm): 180.05 (C-16); 170.36 (C-1); 156.21 (C-9); 136.16; 135.80; 135.55; 133.15; 131.08 (C-5); 130.44 (C-22); 129.36; 129.33; 128.50 (C-11, C-13, C-7, C-19); 128.27 (C-4); 127.89; 127.73 (C-6); 114.49 (C-10, C-14); 67.56 (C-8); 27.24 (C-15); 15.71 (C-15′).

FT-IR(ν, cm^−1^): 3233m (νN-H of amide group); 3094w (νN-H of thioamide group); 2962m (antisymmetric vibration νC-H of methyl group); 2923m (antisymmetric vibration νC-H of methylene group); 2873w; 1675m (νC = O); 1565s; 1511vs (δN-H of amide group); 1364w; 1306m; 1242s (antisymmetric vibration for alkyl-aryl-eter); 1164s (νC = S); 1126m; 1074w; 1045m (symmetric vibration for alkyl-aryl-eter); 978w; 880w; 820w; 757w; 707m; 668w; 611w.

##### 3.2.3.3. 2-((4-Ethylphenoxy)methyl)-*N*-(2-nitro-4-chlorophenylcarbamothioyl)benzamide (1c)

^1^H-NMR (dmso-d6, δ ppm): 12.67 (br s, 1H, NH); 12.14 (br s, 1H, NH); 8.19 (d, *J* = 2.5 Hz, 1H, H-19); 7.93 (d, *J* = 8.8 Hz, 1H, H-22); 7.85 (dd, *J* = 2.5 Hz, *J* = 8.8 Hz, 1H, H-21); 7.63 (br d, *J* = 7.4 Hz, 1H, H-7); 7.60 (m, 1H, H-4); 7.58 (td, *J* = 1.4 Hz, *J* = 7.4 Hz, 1H, H-5); 7.47 (td, *J* = 1.4 Hz, *J* = 7.5 Hz, 1H, H-6); 7.08 (d, *J* = 8.6 Hz, 2H, H-11, H-13); 6.89 (d, *J* = 8.6 Hz, 2H, H-10, H-14); 5.27 (s, 2H, H-8); 2.51 (q, *J* = 7.5 Hz, 2H, H-15); 1.12 (t, *J* = 7.5 Hz, 3H, H-15′).

^13^C-NMR (dmso-d6, δ ppm): 180.64 (C-16); 169.88 (C-1); 156.15 (C-9); 144.26; 136.19; 135.88; 133.29 (C-21); 133.02; 131.34 (C-22); 131.19; 131.14 (C-5); 128.56 (C-11, C-13, C-7); 128.42 (C-4); 127.73 (C-6); 124.54 (C-19); 114.57 (C-10, C-14); 67.41 (C-8); 27.21 (C-15); 15.74 (C-15′).

FT-IR (ν, cm^−1^): 3156m (νN-H of amide group); 3007m (νN-H of thioamide group); 2970m (antisymmetric vibration νC-H of methyl group); 2910m (antisymmetric vibration νC-H of methylene group); 2866w; 1699m (νC = O); 1606w; 1570w; 1504vs (δN-H of amide group); 1469s; 1374w; 1342m; 1286m; 1234s (antisymmetric vibration for alkyl-aryl-eter); 1174s (νC = S); 1156s; 1111m; 1023m (symmetric vibration for alkyl-aryl-eter); 887m; 826m; 788w; 765m; 754m; 734m; 664w; 653m; 594w.

### 3.3. Fabrication of Prosthetic Device Coated with Core/Shell/Adsorption-Shell Nanostructure

**Figure 4:**
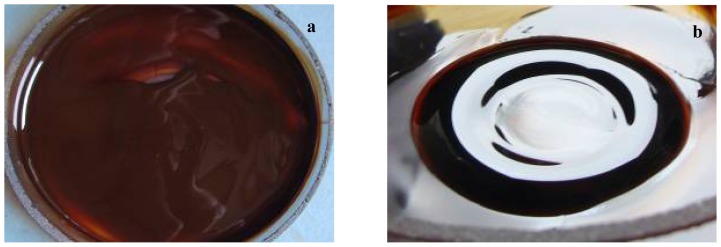
Core/shell/adsorption-shell nanostructure solubilised in chlorofom: (**a**) without magnetic field; (**b**) alligned in magnetic field.

In order to achieve core/shell/adsorption-shell type samples, the adsorption-shell represented by newest synthesized organic compounds (**1a**–**c**) was solubilized in chloroform together with core/shell and grounding until complete evaporation of chloroform. This step is repeated by three times for uniform distribution of the newest organic compounds on the surface of spherical nanostructure. The fabrication was performed by coating the prosthetic device with nanofluid (Figure 4) represented by suspended core/shell/ adsorption-shell in CHCl_3_ (0.33% *w*/*v*).

**Figure 5:**
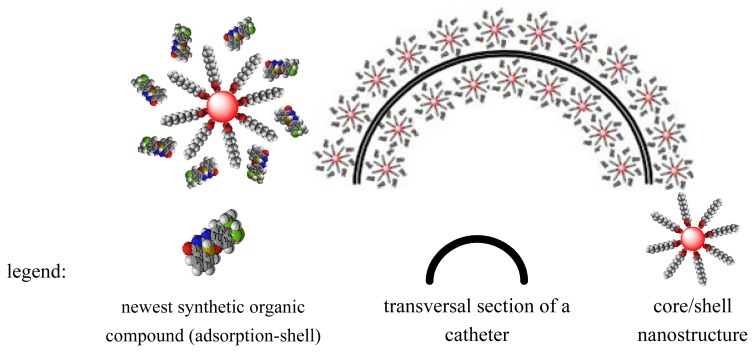
Core/shell/adsorption-shell (**a**); catheter section coated with core/shell/adsorption-shell (**b**).

The layer of core/shell/adsorption-shell on prosthetic device was achieved by submerging the catheter pieces in 5 mL of nanofluid aligned in a magnetic field of 100 Kgf applied for 1 s. Then, the catheter pieces were extemporaneously dried at room temperature. The rapid drying was facilitated by the convenient volatility of chloroform [36]. The coated prosthetic devices were then sterilized by ultraviolet irradiation for 15 min. Figure 5 presents a schematic representation of prosthetic device coated with core/shell/adsorption-shell nanostructure.

### 3.4. Microbial Adherence to the Inert and Modified Prosthetic Devices

The artificial monospecific biofilms were developed using *S. aureus* ATCC 25923 and *Pseudomonas aerugionsa* ATTC 27853 strains. The microbial adherence ability was investigated in 6 multiwell plates, in which there have been placed catheters pieces of 1 cm with and without core/shell/adsorption-shell. Plastic wells were filled with liquid medium, inoculated with 300 μL 0.5 McFarland microbial suspensions and incubated for 72 h at 30 °C. After 24 h the culture medium was removed, the catheters were washed three times in phosphate buffered saline (PBS) in order to remove the non-adherent strains and fresh glucose broth was added. In addition, viable cells counts (VCCs) have been achieved for both working variants (coated and uncoated catheter pieces) at 24 h, 48 h and 72 h, in order to assess the biofilm forming ability of the two strains. The adhered cells have been removed from the catheter sections by vortexing and brief sonication and serial dilutions ranging from 10^−4^ to 1 times the obtained inocula have been spotted on Muller-Hinton, incubated for 24 h at 30 °C and assessed for VCCs [37,38].

### 3.5. Direct Examination of Biofilm Architecture by SEM

In order to evaluate the biofilm formation on coated and uncoated catheters a Scanning Electron Microscopy (SEM) was used. After 24 h of incubation the samples were removed from the plastic wells, washed three times with PBS, fixed with cold methanol and dried before microscopic examination. Samples were visualized by using a HITACHI S2600N electron microscope, at 25 keV, in primary electrons fascicle, on samples covered with a thin silver layer.

### 3.6. Statistical Analysis

The statistical significance of the obtained results was analyzed using *GraphPad Prism* version *5.04* for Windows, GraphPad Software, San Diego California USA. For comparason, we used the number of CFU/mL as revealed by the readings of three values/experimental variant. Logarithmated values were used for statistical analysis. We chose to employ two**-**way ANOVA and Tukey’s multiple comparison tests, for revealing significant differences among the analyzed groups.

## 4. Conclusions

The present study describes an efficient procedure for the synthesis of 2-((4-ethylphenoxy)methyl)-*N*-(substituted-phenylcarbamothioyl)-benzamide. Taken together, our qualitative and quantitative biological assays are clearly demonstrate the efficiency of the core/shell/adsorbtion-shell nanosystem based on magnetite nanoparticles/lauric acid/new benzamides in the design of optimized materials resistant to microbial colonization useful for the further development of functionalized anti-biofilm surfaces with medical applications in the field of catheter-associated infections.

## Figures and Tables

**Table 1:** Characterization data of the new thioureides **1a–c**.

Compound	C%		H%		N%		S%		Molecular weight	Melting point (°C)	Yield (%)

	c.	e.	c.	e.	c.	e.	c.	e.			
1a.	60.13	60.45	4.39	4.27	6.10	6.17	6.98	6.91	459.39	139–141	78
1b.	55.94	56.17	3.88	3.79	5.67	5.62	6.49	6.54	493.83	160–161	81
1c.	58.78	58.59	4.29	4.34	8.94	8.82	6.82	6.89	469.94	144–145	76

where: c = calculated, e = experimental.
